# Comparing the level of physical activity in women with type 2 diabetes

**DOI:** 10.1186/1758-5996-7-S1-A236

**Published:** 2015-11-11

**Authors:** Caroline Hellen Rampazzo Alves, Shirley Aparecida Fabris de Souza, Nathália Caroline Valentini de Azevedo, Décio Sabbatini Barbosa, Danielle Venturini, Alessandra M Okino

**Affiliations:** 1 Universidade Estadual de Londrina, Londrina, Brazil

## Background

Type 2 Diabetes is a condition characterized by blood glucose levels caused by any lack of insulin or the body's inability to utilize insulin effectively. It develops most often in middle-aged and older adults, but may appear in children, adolescents and youth.

## Objective

To evaluate the response of functional capacity and level of physical activity in women with type 2 diabetes.

## Materials and methods

This cross-sectional study consisted of 34 women from the city of Londrina, with age range from 44 to 55 yrs., were divided into two groups: I.DM (patients with Type 2 Diabetes, n=7) and II.Control (normal subjects, n=27). The diagnosis of diabetes was made through a clinical and laboratory evaluation. To assess the level of physical activity was applied the International Physical Activity Questionnaire [IPAQ] and conducted a functional test: Step Test (ST) 2 min to assess the functional status and strength of the lower limbs. Statistical analysis was performed using the Statistical Package for Social Sciences (SPSS-2.0). Statistical significance was accepted for p <0.05. The data numerical correlation of Spearman and Mann-Whitney and chi-square were performed.

## Results

No significant difference between groups I and II in ST (p=0.379) and in the questionnaire (p=0.868). In Group I, 41.7% are inactive and Group II, 56.5% are minimally active (p=0.360). ST was found in a performance at the Group I of 49 (43; 55) repetitions and Group II 45 (38; 50) repetitions (p=0.379). There was a difference between the diastolic blood pressure immediately after the TS groups (p=0.027) and the Borg scale according to claim even after fatigue test (p=0.043). The Results of the IPAQ questionnaire in Group I/Group II respectively were: Active 0%/7.4%; minimally active 57.1%/66.7% and 42.9%/29.4% inactive (p=0.556) and METs 2070/1680 (p=0.868). There was no significant correlation TD and IPAQ (rs=-0.236/p=0.180). In Group I all have the diagnosis of metabolic syndrome in Group II 63%.

## Conclusion

Type 2 Diabetes is strongly related to metabolic risk, we found that although not significant was no difference in performance between groups. Physical activity brings metabolic and cardiovascular benefits and should be encouraged to minimize risk factors.

